# Ovarian Torsion after Hysterectomy: Case Report and Concise Review of the Reported Cases

**DOI:** 10.1155/2018/6267207

**Published:** 2018-07-04

**Authors:** Demetrio Larraín, Andrés Casanova, Iván Rojas

**Affiliations:** Department of Obstetrics and Gynecology, Clínica Santa María, Santiago, Chile

## Abstract

Ovarian torsion after hysterectomy is a rare event. The diagnosis of ovarian torsion is challenging because symptoms are nonspecific. We present a case of ovarian torsion 2 years after laparoscopic hysterectomy (LH). Furthermore, we performed a literature review about ovarian torsion after hysterectomy. This case shows that, in cases of acute onset pelvic pain in patients with history of hysterectomy, the adnexal torsion must be kept in mind in the differential diagnosis, especially in those women who had undergone LH.

## 1. Introduction

Ovarian torsion accounts for 2-3% of all acute gynecological emergencies. It remains a clinically difficult diagnosis as the symptoms are usually nonspecific. Ovarian torsion can occur at any age and also after hysterectomy [1]. Although hysterectomy with ovarian conservation is not a risk factor for torsion [1], it seems to be more frequent after laparoscopic hysterectomy [2]. Therefore, despite it is a rare event, its prevalence could increase in the future with the widespread use of laparoscopic approach. We present a case of ovarian torsion after a laparoscopic hysterectomy and performed a literature review about reported cases.

## 2. Case Presentation

**Figure 1:**
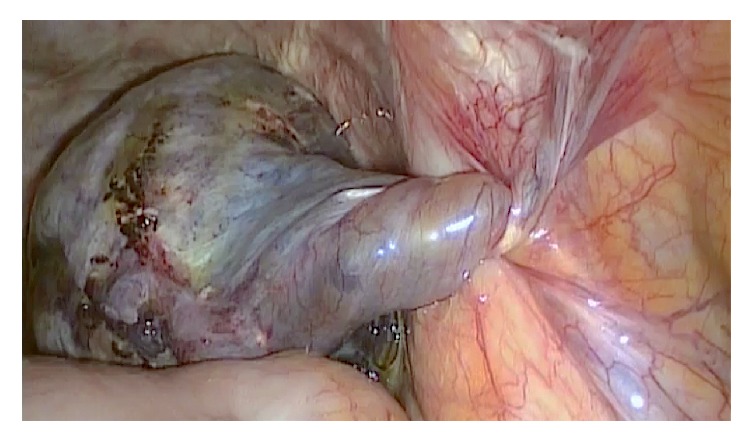
Laparoscopic view of a twisted right ovary. Note the absence of adhesions, which may have facilitated torsion.

A 41-year-old woman, gravida 3, para 3, was admitted to our institution with a 12-hour history of acute onset pelvic pain, nausea, and vomiting. She had undergone total laparoscopic hysterectomy 2 years previously. The abdominal exam revealed mild distention and tenderness over the right lower quadrant. Vaginal examination revealed exquisite pain in the right vaginal fornix and the finding of a painful adnexal mass in the rectovaginal pouch of Douglas. Transvaginal ultrasonography showed a 60-mm cystic lesion in the right ovary with moderate ascites. We performed an exploratory laparoscopy and found a right adnexal torsion (Figure 1) and a right adnexectomy was successfully performed. Since the left ovary was normal a left ovariopexy was also performed.

## 3. Discussion

Ovarian torsion after hysterectomy is a rare event with a prevalence of 7.91/1000 hysterectomies [2]. Although hysterectomy is not a risk factor for ovarian torsion [1], it has been estimated that approximately 8% of adnexal torsions occur in patients with previous hysterectomy [3, 4]. To date, there are no data on how different hysterectomy techniques may affect the risk of future ovarian torsion. However, several cases of ovarian torsion have been reported after laparoscopic hysterectomy (LH) (Table 1), while to our knowledge, only one case has been published after abdominal approach [6]. This could be explained by the fact that laparoscopic approach has been associated with both fewer postoperative adhesions [7] and less adhesion-related complications [8] when compared to laparotomy, in both gynecologic and pelvic surgery. The latter could be a direct consequence of the lesser peritoneal trauma and less inflammatory response during laparoscopy [9, 10]. Moreover, our technique of LH [11] includes a wide fenestration of the broad ligament, which is left open after surgery. Based on our observations, the ovaries remain much more movable after LH when compared to open approach (due to the skeletonization of infundibulopelvic ligament). For that reason, we perform prophylactic oophoropexy after hysterectomy only when the infundibulopelvic ligament has been excessively skeletonized and the ovaries remain too much mobile. However, in agreement with other authors [12, 13], we perform systematic oophoropexy in cases of recurrent torsion, excessive length of utero-ovarian ligament, torsion of a solitary adnexa, or contralateral pexy in case of adnexectomy of the twisted adnexa.

This case shows that, in cases of acute onset pelvic pain in patients with history of hysterectomy, the adnexal torsion must be kept in mind in the differential diagnosis, especially in those women who had undergone LH. In addition, we encourage that, during LH with ovarian conservation, the fenestration of the broad ligament should be performed in the direction of the uterine artery and not towards the infundibulopelvic ligament, in order to keep the ovary more fix to the pelvic sidewall.

## Figures and Tables

**Table 1:** Ovarian torsion after hysterectomy.

Author, year [Reference]	Cases	Time from hysterectomy	Type of hysterectomy	Symptoms
Mashiach, 2004 [2]	7	2.64 years	Laparoscopic	Pelvic pain

Houry, 2001 [3]	7	NA	NA	NA

Lo, 2008 [4]	5	NA	NA	NA

Ciebera, 2016 [5]	1	7 months	Laparoscopic supracervical	Asymptomatic suspicious pelvic mass

Elhjouji, 2015 [6]	1	4 years	Abdominal	Pelvic pain

NA: not available.
